# The effect of generation change on the accuracy of full arch digital impressions

**DOI:** 10.1186/s12903-023-03476-z

**Published:** 2023-10-18

**Authors:** Judit Schmalzl, Ivett Róth, Judit Borbély, Péter Hermann, Bálint Vecsei

**Affiliations:** Department of Prosthodontics, Semmelweis University, Szentkiralyi street 47, Budapest, 1088 Hungary

**Keywords:** Accuracy, IOSs, Full arch, Trueness, Precision, Inter-operator reliability

## Abstract

**Purpose:**

This study is aimed to evaluate the effect of generation change on accuracy of IOSs on full-arch scans and the inter-operator reliability.

**Methods:**

In this study, 6 different IOS were tested: 3Shape Trios 3 (20.1.2.), 3Shape Trios 4 (20.1.1.), Medit i500 (2.3.6.), Medit i700 (2.4.6.), Planmeca Emerald (6.0.1.) and Planmeca Emerald S (6.0.1.). Eighteen dental students, inexperienced in scanning, took part in this study as operators. Each operator made 10 digital impressions; altogether, 30 impressions were made by each scanner. The 30 STL files were imported to the Geomagic Control X program, where they were compared to a reference STL file; the surface point’s deviation of the full arch and the distance between the second molars’ distobuccal cusps were measured, the inter-operator reliability was also investigated.

**Results:**

A significant increase in accuracy was found between Trios 3 and 4 in the case of both parameters and between Medit i500 and i700 in the case of full arch. There was no significant difference between Planmeca generations. In case of the inter-operator reliability no significant difference was detected.

**Conclusion:**

Within this current study’s limitation, it can be concluded that surface digitalization’s accuracy can be modified with generation changes and that digital technology is less technique sensitive than traditional impression taking.

## Introduction

Well-known instruments for taking optical impressions in dentistry are intraoral scanners (IOSs). A light source is projected onto the dental arch by the IOS device, and images of the tissues are captured by the imaging sensors and processed by the scanning software, which generates a point cloud [1, 2]. The accuracy of digital impressions is one of the most important factors that determine the long-term success of final restorations [3]. Accuracy (trueness and precision) reflects close agreement between a test result and an accepted reference value [4]. Precision reflects the variability between repeated measurements of a single sample, while trueness quantifies how well a given measure correlates with actual values [5]. Based on the literature, the accuracy of IOSs exceeds the accuracy of traditional impressions, although digital impression-taking still has limitations. For short-span restorations up to a quadrant, IOSs produce a more accurate impression than conventional restorations [6–8].

Several factors can influence the accuracy of digital impressions. According to the literature, accuracy is greatly affected by the scanning strategy – the order in which the surfaces of teeth are digitalized – and the operator’s experience [9]. The scanning sequence has an especially great influence in the case of full arch scans [10, 11]. It has been stated in previous studies that the accuracy of the IOS shows a reduction as more teeth are captured [12–15]. Most IOSs generate a 3D virtual model by capturing 2D images and stitching them together with overlaps. Stitching errors can be compounded and result in more significant inaccuracy in the case of full arch impressions [16]. The presence of edentulous ridges also impacts the accuracy due to the lack of clear and individual geometric information for optical scanning. [17]. Reflection from the metal surfaces (e.g. restorations or orthodontic brackets), intense saliva flow, or limited access to the oral cavity (degree of mouth opening) can negatively impact the sharpness and resolution of recorded images, thereby affecting the accuracy of the digital impression [1, 18–21].

IOSs are continuously developing, and manufacturers are developing new software and new generations of scanners to provide properties such as accuracy, ergonomic design, speed, and efficiency. New software updates have additional features and promise smoother, more stable optical mapping and more accurate digital impressions.

In the literature, previous studies have evaluated the effect of different software or software functions on accuracy [22–24]. In 2020, Chiu et al. [25] measured the effect of a new software feature (high resolution) to 3Shape Trios 3 IOS. In that study, there was no significant difference between the default resolution and high resolution in terms of accuracy, although the scanning time and the number of captured images/scans were significantly different. In research in 2023 provided by the Department of Prosthodontics, Semmelweis University, the effect of software updates on accuracy was measured and was found to significantly impact trueness and precision [26]. Another study also investigated the influence of software updates and found negative and positive impacts [27].

The generation change of IOSs meant that the manufacturer created a brand-new IOS (new hardware background) that worked with new software [2]. Based on present literature, there is little information about the influence on accuracy of the generation change of IOSs [28]. Knowing the difference in accuracy between the generations of IOS is important for long-term clinical application and can help dentists choose appropriate devices for their indication area.

This study aimed to evaluate the effect of generation change on the accuracy of IOSs on full-arch scans and to investigate the inter-operator reliability. Hence, the first null hypothesis is that there is no significant difference between the accuracy of the old and new generation IOSs. The second null hypothesis is that there is no significant difference between the operators regarding the accuracy of the digital impression.

## Material and method

Six different IOS were evaluated in this study: 3Shape Trios 3 (software version: 20.1.2.), 3Shape Trios 4 (software version: 20.1.1.), Medit i500 (software version: 2.3.6.), Medit i700 (software version: 2.4.6.), Planmeca Emerald (software version: 6.0.1.) and Planmeca Emerald S (software version: 6.0.1.). From each manufacturer, two different generations of scanners were investigated: the previous and latest generations available on the dental market at the time of this study (Table 1). The 3Shape intraoral scanners use confocal laser scanning technology, Medit and Planmeca scanners use the principle of triangulation to create the virtual model with video recording method [29, 30]. It is a well-known fact for each intraoral scanners that the new generations of the devices have more special properties (e.g., individual movement detection, smile design, denture workflow etc.) than the previous versions, therefore, it was true for the IOSs which were used in our study [28]. Besides that, there were further differences between the generations of the examined IOSs. In case of Trios and Medit the difference was the configuration of the devices: the previous versions (Trios 3 and Medit i500) are wired, and the new generations (Trios 4 and Medit i700) are wireless IOSs. Furthermore, initially when the Trios 4 was dropped to the dental market, it had an additional scanner tip which could be used for caries detection. Later, the manufacturer company made a software and hardware update: with this development the newly manufactured devices (not just the Trios 4, but also Trios 3 IOSs) were able to detect caries without any special tip [31]. In case of Planmeca IOSs the main differences between the versions are the tooth shade selection and the caries detection [28, 32]. These mentioned special properties depends on the hardware background of the devices, therefore, they become available after the hardware developments [28].

**Table 1:** The scanners used in this study

Manufacturer	Hardware	Software	Release date
3Shape	Trios 3	20.1.2.	2015
3Shape	Trios 4	20.1.1.	2019
Medit	i500	2.3.6.	2018
Medit	i700	2.4.6.	2021
Planmeca	Emerald	6.0.1.	2017
Planmeca	Emerald S	6.0.1.	2019

The reference model was a polymethyl methacrylate (PMMA) maxillary model with supragingival prepared teeth (FDI World Dental Federation) included numbers 11, 14, 17 for a crown and 26 for an inlay; teeth 15 and 16 were missing.

The reference data set was created by an industrial scanner (AICON SmartScan-3D C5; AICON 3D Systems GmbH, Braunschweig, Germany) with an 8 μm accuracy according to the manual guide [33]. Eighteen operators took digital impressions with the 6 IOS devices (3 operators of each IOS). With each IOS, 10-10-10 virtual models were made: 10 by each operator, resulting in 30 STL files per IOS (altogether 180 impressions). This sample size was defined based on a previous study with an effect size of 1.0, α = 0.5 and a power of 0.80 [27]. The operators were dental students in their 6th or 10th semester of dental education and had no experience in intraoral scanning. Before taking the optical impressions, theoretical and practical education was provided about using the current IOS held by the distributor company of Hungary [28].

The scanning strategy was determined by the manufacturer of each scanner. The accuracy of virtual models created by the IOS depends on the scanning strategy, therefore knowledge of the scanning sequence is crucial [14, 34, 35]. In the case of Trios IOS, the suggested scanning path is as follows: the upper and lower jaws both should be started at the occlusal surface. When scanning the maxilla, starting from the occlusal to the buccal to the palatal surface is recommended, and when scanning the mandible, from the occlusal to the lingual to the buccal surface is preferred [36–39]. In the case of Medit IOS, the recommended scanning strategy starts at the occlusal surface (in both upper and lower jaws), and then the scanning should be continued on the oral surface and finally the vestibular side [40]. Using the Planmeca Emerald IOS, the scanning strategy is as follows (both in maxilla and mandible): the scanning starts on the occlusal surface from the molar region until the middle of the arch, after that the oral and then the vestibular surface is scanned. In the next step, the same path is repeated on the opposite side of the arch [41]. During the measurements, the operators were assisted by a supervisor with more than 5 years of experience in intraoral scanning. Each IOS device was calibrated before scanning according to the user’s guide.

**Fig. 1:**
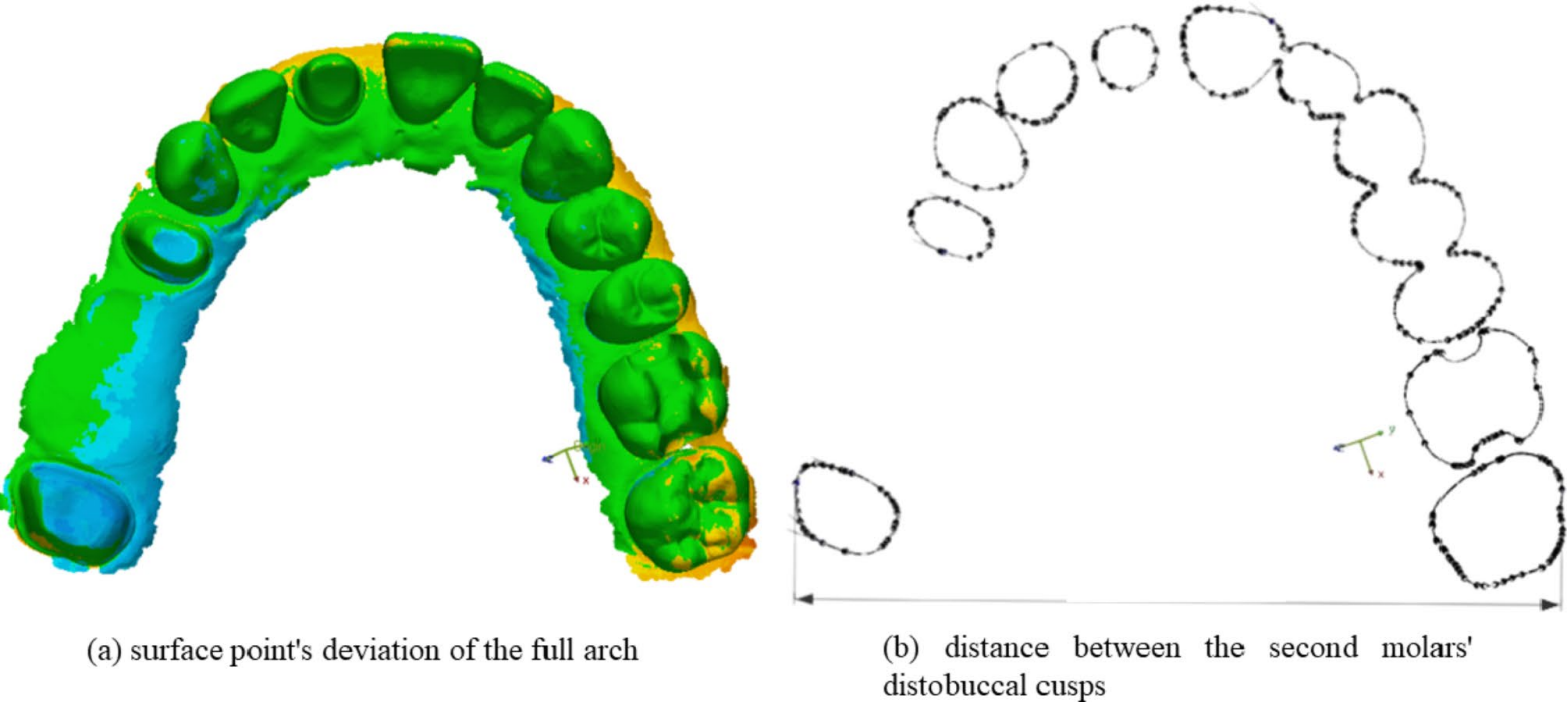
Measured parameters: (**a**) surface point’s deviation of the full arch (WHL), (**b**) distance between the second molars’ distobuccal cusps (arch distortion)

The STL files were imported into the Geomagic Control X program, and all unnecessary parts were cropped (such as the tuber maxillae and the palate) to make them uniform. The reference and measured datasets were superimposed, and distortions were calculated. The surface point deviation of the full arch (WHL) and the distance between the distobuccal cusps of the second molars were measured (Fig. 1). This showed the variation between the digital impressions made by the examined IOS and the reference dataset. Additionally, the inter-operator reliability was also investigated.

Surface point deviation and absolute arch distortion data were described in terms of median and interquartile range (IQR) and visualized using box-whisker plots. Multilevel mixed-effects linear regression models were used to derive estimates of new versus old generation difference in the outcome (surface deviation or arch distortion, both log-transformed to improve normality) for each IOS brand. The model recognized the non-independence of repeated measurements by the same operator and allowed heteroscedastic residuals across IOSs. Between-operators heterogeneity was visualized using box-whisker plots and assessed through the estimate of the operator-level variance term and its standard error. The statistical package Stata was used for data handling and analysis. Statistical significance was defined as p < 0.05.

## Results

**Fig. 2:**
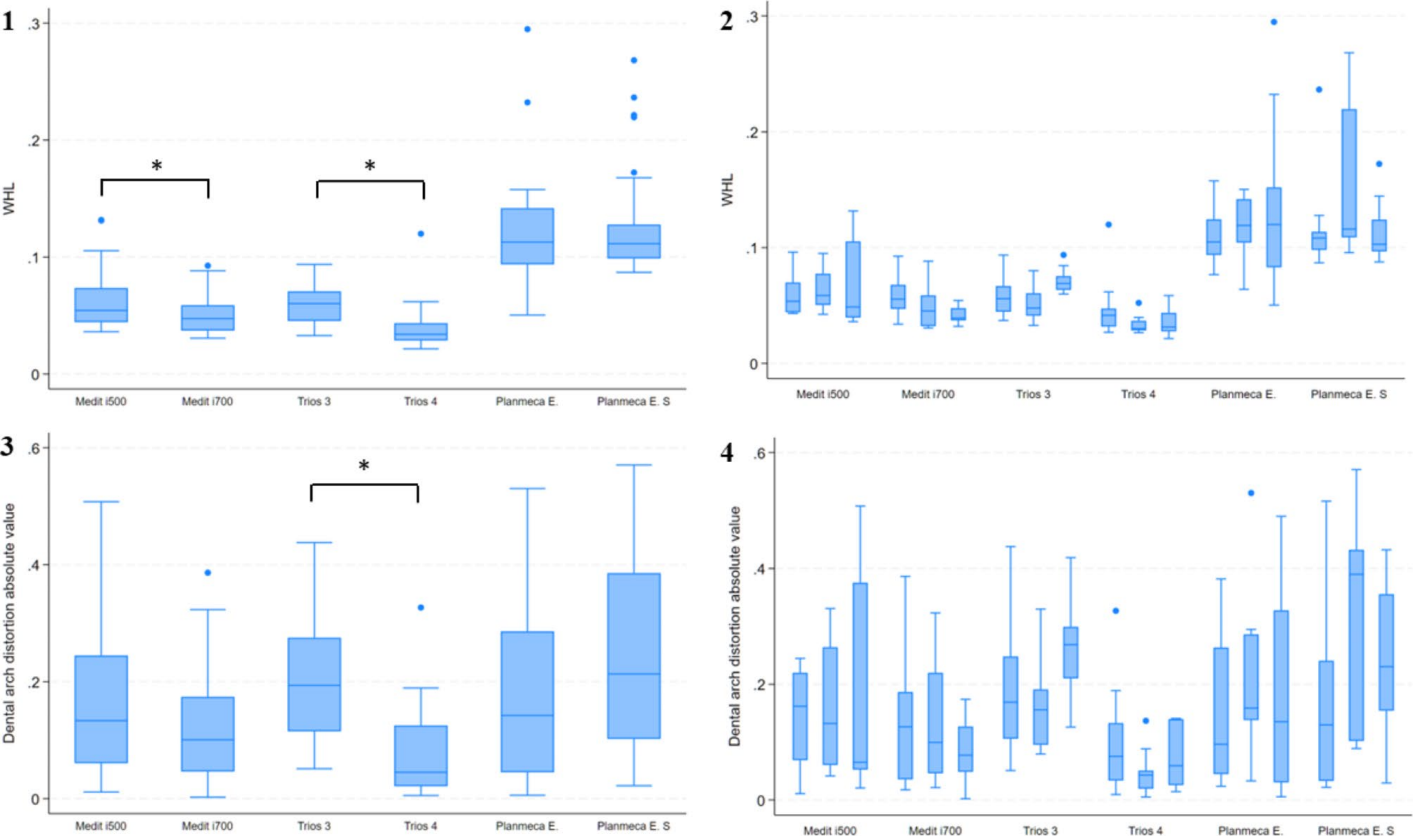
(**1**) results of WHL (**2**) results of inter-operator reliability in case of WHL (**3**) results of arch distortion (**4**) results of inter-operator reliability in case of arch distortion; *significance (p ≤ 0.015)

The results are presented in the form of median [IQR]. For the surface deviation of the full arch 3Shape Trios 4 had the highest trueness in the current study at 34.0 [14.8] µm. For the other IOS, the trueness results were as follows: 3Shape Trios 3, 60.2 [25.3] µm; Medit i500, 54.4 [29.2] µm; Medit i700, 47.3 [21.7] µm; Planmeca Emerald, 112.8 [48.1] µm; and Planmeca Emerald S, 111.5 [29] µm. The accuracy of Trios 4 was significantly better than the previous generation’s results. The Medit i700 also produced significantly more accurate impressions than the Medit i500. In the case of Planmeca scanners, the generation change did not affect the accuracy (Table 2; Fig. 2).

**Table 2:** Table of results

Scanner	WHL		Arch distortion (absolute values)	
	median	IQR	median	IQR
Medit i500	54.4 μm	29.2 μm	133.2 μm	184.1 μm
Medit i700	47.4 μm	21.7 μm	100.6 μm	127.8 μm
Trios 3	60.2 μm	25.3 μm	193.5 μm	160.2 μm
Trios 4	34 μm	14.8 μm	45 μm	103.9 μm
Emerald	112.8 μm	48.1 μm	142.2 μm	241.1 μm
Emerald S	111.5 μm	29 μm	213.1 μm	283.6 μm

In case of arch distortion, the absolute values were calculated. The measured data were as follows: Trios 3, 193.5 [160.2] µm; Trios 4, 45 [103.9] µm; Medit i500, 133.2 [184.1] µm; Medit i700, 100.6 [127.8] µm; Planmeca Emerald, 142.2 [241.1] µm; and Planmeca Emerald S, 213.1 [283.6] µm. In this parameter, we only found significant improvement in accuracy between the Trios generations. In the case of Planmeca and Medit IOS, no significant difference was detected (Table 2; Fig. 2). According to these results, the first null hypothesis was partially rejected.

In case of the inter-operator reliability, we did not find significant difference between the operators regarding the accuracy of the digital impressions (Fig. 2). Therefore, the second null hypothesis was accepted.

## Discussion

Software updates and new generations tend to improve IOS performance in terms of accuracy. Based on our results, not all new IOS generations fulfil these requirements and ideas. In this study, the accuracy of 2 pairs from 3 manufacturers, overall 6 IOSs were examined: Trios 3 (20.1.2.) and Trios 4 (20.1.1.) from 3Shape, i500 (2.3.6.) and i700 (2.4.6.) from Medit, and finally Emerald (6.0.1.) and Emerald S (6.0.1.) from Planmeca.

There is little information in the literature about the accuracy of the latest generation of IOS devices. Most previous studies use edentulous models or a reference model with implants; therefore, they are not comparable with our results [42, 43]. In contrast, Trios 3 IOS was introduced in 2015, Medit i500 IOS was established in 2018, and Planmeca Emerald was shown in the dental market in 2017 [2, 44]. Numerous studies have investigated the previously mentioned IOSs [45–48]. In 2019 Michelinakis et al. [46] investigated the accuracy of the full arch with Trios 3 (1.6.9.1), Medit i500 (2.3.0) and Planmeca Emerald (5.3.2.13). According to their data, the following trueness was measured: Trios 3, 16.8 ± 3.8 μm; Medit i500, 15.8 ± 5.9 μm; and Planmeca Emerald, 56.5 ± 15.2 μm. There are some differences between the results from the mentioned publication and our measurements, but it is important to highlight that our results match the order of the scanner versions: Medit i500 had higher trueness than Trios 3, and Planmeca Emerald proved to be the least accurate in our study. In 2021, Nulty [49], compared the accuracy of full arch digital impressions of nine IOSs, including Trios 3, Trios 4, and Medit i500 on full arch. Trios 4 (20.8 ± 6.2 μm) proved to be more accurate than Trios 3 (27.7 ± 6.8 μm). This result is comparable with our evaluation. The results the present study are not in agreement with those of the studies by Michelinakis et al. [46] and Nulty [49], which may be due to difference between the type of the reference model (their model did not contain an edentulous ridge or prepared teeth, which can affect the accuracy negatively, as mentioned above). Park et al. [16] investigated the Trios 2 and Trios 3 IOS in 2019: on full arch, the new generation scanner (Trios 3) produced higher accuracy than the old generation scanner (Trios 2). In 2022, Ochoa-López et al. [50] evaluated the accuracy of Medit i500 and i700 IOS, among others. The results show a slightly increased accuracy in the case of Medit i700. These studies could also support the statement, similar to our results on full arch, that the generation change positively impacted accuracy.

The accuracy of intraoral scans depends on many factors. One of them is the efficiency of the operator (who takes the digital impression) [9]. Dental students without experience may make mistakes and inaccurate impressions. We did not find significant differences between the accuracy of virtual models made by several dental students (operators). It can be concluded that digital technology, which tries and promises to be a more accessible, reliable, and not overly technique-sensitive alternative to conventional impression-taking, should be important for teaching dental students. In 2017, Kamimura et al. [51] found that digital impression-taking yielded superior reproducibility compared to conventional impression techniques and was not affected by the operators’ experience. According to these studies, IOSs are less technique sensitive than traditional impression-taking methods.

Further studies should examine whether experts and students have significant differences based on accuracy in IO scanning. It would be interesting to determine the performance difference between the two groups in the conventional and digital impression procedures.

Our study has some limitations that should be mentioned. This was an in vitro study. The circumstances were more ideal than a real clinical situation, without saliva or the movement of the tongue or the patient’s head; these factors can negatively impact the quality of the digital impression. The operators were dental students who did not use IOSs before. It is known that efficiency is an influencing factor regarding accuracy as well as scanning technique. Furthermore, it would be also important to investigate the accuracy using different types of models, such as edentulous models and crowded dentition. In this study, IOSs from only three different manufacturers were measured. It is important to evaluate other types of IOS as well.

## Conclusion

Despite this current study’s limitations, it can be concluded that the accuracy of surface digitalization can be modified with generation changes. In the case of Trios, the new generation obviously provides a more accurate digital impression than the previous generations. In the case of Medit scanners, the generation change causes slightly better accuracy. On the other hand, the generation change did not affect accuracy in the case of Planmeca intraoral scanners. Furthermore, digital technology is less technique sensitive than traditional impression taking.

## Data Availability

The datasets used and/or analyzed in the current study are available from the corresponding author upon reasonable request.
